# Research on factors influencing Chinese parents’ support for death education: a cross-sectional survey

**DOI:** 10.3389/fpubh.2024.1285208

**Published:** 2024-02-28

**Authors:** Hejie Chen, Yuan Xiao, Xincheng Huang, Siyuan Fan, Haiwen Wu, Linxiao Li, Yibo Wu

**Affiliations:** ^1^School of Economics and Management, Beijing Institute of Graphic Communication, Beijing, China; ^2^Blockchain Research Institute, Renmin University of China, Beijing, China; ^3^Department of Preventive Medicine, Yanjing Medical College, Capital Medical University, Beijing, China; ^4^School of Public Health, Peking University, Beijing, China

**Keywords:** death education, Chinese parents, supportive attitudes, cross-sectional survey, ecological systems theory

## Abstract

**Objective:**

This study aims to explore the factors influencing Chinese parents’ attitudes toward death education. Given the current lack of such education in China, this research is particularly significant. Death education is vital for shaping the values of young people and alleviating mental health issues, such as depression and suicidal tendencies. By identifying these influencing factors, this study seeks to provide guidance for policymakers and educators in promoting the development and widespread adoption of death education.

**Methods:**

To do so, a national cross-sectional quota sample of 12,435 Chinese parents was used. Borrowing from social-ecological theory, the researchers carried out multiple stepwise regression analyses to examine the individual, family, and social-level factors that shape the supportive attitudes of Chinese parents toward death education.

**Results:**

The findings revealed that at the individual level, parent (*β* = 0.04, *p* < 0.001), education level (*β* = 0.07, *p* < 0.001), and religious belief (*β* = −0.02, *p* < 0.05) were significant predictors of Chinese parents’ support for death education. Meanwhile, at the family and social level, average monthly household income (*β* = 0.07, *p* < 0.001), family health (*β* = 0.03, *p* < 0.05), family communication (*β* = 0.02, *p* < 0.05), social support (*β* = 0.15, *p* < 0.001), neighborhood relations (*β* = 0.11, *p* < 0.001), and social network size (*β* = 0.05, *p* < 0.001) were significant predictors of Chinese parents’ supportive attitudes toward death education.

**Conclusion:**

Based on these findings, it is suggested that the relevant development, planning, publicity, and public welfare groups and government departments should promote death education, provide more social support, and encourage neighborhood harmony. As higher education and average monthly household income were found to significantly impact the support, the government should improve access to higher education and actively work to increase residents’ income to facilitate the development of death education.

## 1 Introduction

Rapid social and economic development have placed adolescents under increasing competitive pressure, thereby increasing the frequency of social disengagement and even suicide, which is now a leading cause of death among adolescents worldwide (1). In China alone, around 3,000 adolescents commit suicide each year (2). The increasing number of deaths demonstrates that adolescents are now more vulnerable to negative emotions such as anxiety and fear than previous generations. In addition, due to the notable ascent of death education in the education system, there is a lack of basic understanding or consideration of death among adolescents, thus increasing the likelihood they may resort to suicide ideation (3, 4). Research shows that children have a preliminary understanding of death at an early stage of their development (5). For instance, research has shown that after the age of 7–8, children’s perception of death will be more practical and objective (6). If children cannot be properly guided at this stage, it is likely that they do not properly understand death, which may lead them to develop negative emotions, affecting their whole life (7). Therefore, death education is vital for the physical and mental development of children and adolescents: it will improve how they comprehend the world around them as grow (8) and help them to form a correct view of life and death (9).

Death education originated in the United States in the 1920s, later reviving government promotion starting in the late 1950s. Over the next decade, the number of schools, hospitals, and social service institutions providing death education increased to over 2,000 (10–12). Subsequently, countries such as Japan, the United Kingdom, and Australia also began to actively carry out death education to help individuals form a correct view of death, comprehend the cycle of life and death, and accordingly appreciate the fleeting beauty of life (13). In China, due to the influence of traditional culture, people tend to avoid death-related topics. As a result, death education has not received corresponding attention. It was not until the late 1980s that Chinese scholars began to pay attention to death education, and the government and social organizations began to emphasize its promotion. Unfortunately, to date, there are only around 20 universities in China that offer death education courses, most of which are theoretical and lack any practical guiding meanings. It should also be highlighted that the inclusion of death education in primary and secondary school curricula is seriously lacking (14, 15). Schools that fail to provide death education are not furnishing their students with lifelong education. Therefore, it is necessary to integrate death education into school teaching (16, 17). To do so, many factors need to be considered to build a death education system; vital among these is parental support for death education, which influences the actual teaching to some extent (18, 19). With this in mind, Chinese parents need to be considered in any attempt to tackle this issue. At present, most research on death in China largely focuses on the basic theory and practice of death education at both the policy and social levels (20), with very little research exclusively addressing the role of parents. To attend to this research gap, the present paper will study the degree of parents’ support for death education in China and its influencing factors; doing so will provide evidence to support the inclusion of death education in the education system.

## 2 Theoretical basis and research hypothesis

The ecological systems theory studies the interactions between human behavior and the surrounding environment in which it is carried out. It specifically focuses on the social environments for human growth and survival, such as family, school, and society, which together form a social ecosystem. It emphasizes the importance of the social ecological environment for analyzing and understanding human behavior, underscoring the notable impact that the interaction between systems and individuals has on it (21). The ecological systems theory was first proposed by American psychologist Bronfenbrenner. He divided the ecological system into four different systems—the microsystem, the mesosystem, the exosystem, and the macrosystem—emphasizing the “person-in environment” concept (22, 23). The microsystem refers to the physiological, social, and psychological systems of an individual within the context of a given social ecological environment. The mesosystem is constituted by the social factors that directly impact individuals, such as family, friends, and colleagues. The exosystem refers to those factors that influence individuals in the mesosystem, such as their parents’ work environments. Finally, the macrosystem is broader than the mesosystem, including culture, society, and institutions. Based on Bronfenbrenner’s work, Zastrow further developed society ecosystems theory: he embarked on a deeper analysis of the multi-layered system made up of human behavior and social environment. He then divided individual social ecosystems into three types: micro systems, mezzo systems, and macro systems (21).

There are many factors that influence the support of Chinese parents for death education. This paper draws on previous research, the definitions of each system outlined under society ecosystems theory, and Zastrow’s classification method to explore and analyze this issue from the micro, mezzo, and macro levels, respectively.

At the micro-individual level, age, parent, educational background, partner status, and religious beliefs may be important factors that influence Chinese parents’ support for death education. Aging increases the frequency with which parents encounter death, forcing them to grapple with the concept (24). Those parents that have a more comprehensive cognition of death and a calmer attitude are likely to understand the role of death education in helping their children to recognize and manage the negative emotions stemming from death (25), resulting in higher support for death education. It is interesting to point out that women are more anxious and fearful about death than men (26–28), and therefore, have more demand for death education (29). This difference in attitude might lead mothers to exhibit higher levels of support for their children’s death education. The highest level of education an individual has achieved may also be an important factor: the more educated an individual is, the more they understand death, resulting in less negative feelings toward death as a concept (29, 30). Parents are more willing to help their children correctly conceptualize death through death education to minimize any negative feelings. Moreover, partner status may also influence Chinese parents’ support for death education. As parents with partners generally have better life experiences and higher happiness (31–33), and are more positive about death (34, 35), they may be more supportive of their child receiving death education. Furthermore, religious beliefs may also be a factor, as religions often provide reasonable and positive explanations for death, such as reincarnation or the idea that death forms a part of a god’s broader plan (36). Chinese parents who have religious beliefs may have more positive attitudes toward death (37), and therefore, are more willing to expose their children to positive views about death through death education.

At the mezzo-family level, average monthly household income is an important factor influencing Chinese parents’ support for death education. In general, a higher average monthly household income suggests that parents are better educated and are more likely to have been exposed to different cultures and ideas (38). In this way, they tend to adopt a more open and diverse view of death, as opposed to exclusively seeing it as a taboo or scary subject. In addition, these parents are also more likely to understand the importance and value of death education and support its provision in schools (39). It should also be underscored that family health is an important factor: family health here refers to the daily living status of family members and the health resources available. It includes communication, interaction, and support among family members, family lifestyle and behavior habits, family health care and prevention measures, family’s ability to cope with stress and crises, and family participation in community activities and resource utilization (40). Where the entire family is in good health, the parents will actively support their children’s development (41), including supporting death education to improve their children’s ability to handle difficult situations (42). Family communication is also a central influencing factor. Family communication is the process through which family members express and understand each other in various ways. This broad process is mediated by the individuals’ surroundings and backgrounds (43). Family communication includes daily conversations, conflict resolution, decision-making, and emotional expression that the place between family members. It has been shown that family communication relating to death will affect family members’ emotional responses and long-term mental health (44). If family communication is smooth, open, and positive, parents are more likely to establish a sense of trust and understanding with their children. Additionally, when parents demonstrate a willingness to talk about sensitive topics, such as death, with their children, they can provide the appropriate guidance and support (45). Contrastingly, where family communication is poor, closed, and negative, parents are more likely to deny or refuse to engage with topics such as death, which will hinder children’s understanding and acceptance of death.

At the macro-society level, social support is an important factor affecting Chinese parents’ support for death education. In this context, social support refers to the emotional, informational, material, or other forms of support an individual receives from individuals or groups to help deal with the pressures and difficulties that arise in daily life (46, 47). Social support can be emotional, informational, or instrumental, all of which enhance parents’ understanding of and attitude toward death education, while also improving their willingness to support their children’ death education (46). Neighborhood relationships are another important factor. Neighborhood relationships embody the interactions and connections between individuals and other residents in their living environment, including trust, participation, and sharing (48). Neighborhood relationships mediate how parents engage with the topic of death and create an atmosphere in which death can be freely discussed, ultimately promoting the parents to change their attitude toward death. Hence, it can be seen that harmonious neighborhood relationships can improve parental support for death education (48).

The social network scale is another vital factor that refers to the number of people that an individual contacts or interacts with within a determined period of time. This measure is used to reflect the position and scope of an individual in society (49). Parents with larger social networks have richer experiences related to death; it is expected this will encourage them to think more deeply about death, engage in discussions on the subject, form a more comprehensive understanding of it, and thus exhibit greater support for death education.

Accordingly, the following hypotheses are proposed in this study:

At the micro-individual level:

*H1* (Age): The older the parents are, the more supportive they will be of death education.

*H2* (Parent): Mothers are more supportive of death education than fathers.

*H3* (Education level): Support for death education is higher among more highly educated parents compared to parents with a junior high school education or below.

*H4* (Partner status): Partnered parents are more likely to support death education than non-partnered parents.

*H5* (Religious belief): Religious parents are more likely to support death education than non-religious parents.

At the mezzo-family level:

*H6*: The higher the average monthly household income, the more supportive parents are of death education.

*H7*: The healthier the family, the more supportive parents are of death education.

*H8*: The better the family communicates, the more supportive parents are of death education.

At the macro-society level:

*H9*: The stronger the degree of social support, the more supportive parents are of death education.

*H10*: The more harmonious the neighborhood, the more supportive parents are of death education.

*H11*: The larger the social network scale, the more supportive parents are of death education.

## 3 Methods

### 3.1 Procedures

This study utilized cross-sectional data collected using multi-stage sampling techniques between June and August 2022. Quota sampling was employed to select subjects from 120 Chinese cities based on attributes including parent, age, and urban/rural area. Thus, the data sample used in this study conforms to the pyramid structure of China’s population, reflects the composition of China’s population, and is well represented (50). The survey team, recruited and trained interviewers from each survey city. Each city was assigned at least one interviewer or interview team: each interviewer was responsible for administering 30–90 questionnaires, while each interview team was responsible for administering 100–200 questionnaires. The survey was conducted using Questionnaire Star, a survey tool commonly used in China. The interviewers provided the subjects with a link to the questionnaire for them to fill out in their own time. For those older adults who struggled to properly complete the answers, the interviewers assisted in filling out the questionnaires offline on a one-on-one basis. Prior to commencing the questionnaire, each participant was required to record their informed consent.

### 3.2 Participants

Inclusion criteria: (1) Participants with children; (2) Nationality of the People’s Republic of China; (3) Part of China’s permanent resident population with an annual travel time ≤ 1 month; and (4) Understands the meaning of each questionnaire item.

Exclusion criteria: (1) Persons with unconsciousness or mental disorders; (2) Participation in other similar research projects (To reduce the interference of repetitive samples or prevent research fatigue).

This study employed a multistage sampling approach to ensure the representativeness of the sample. Sampling encompassed 23 provinces, five autonomous regions, and four municipalities across the country, with the sampling ratio determined based on the population proportion from the seventh national census data. At least 500/1,000/1,500/2,000/2,500 individuals were sampled from each province/autonomous region/municipality, estimating a total sample size of 20,000. The sampling process included levels from municipal, district, county, township/town, and community/village, along with individual-level sampling based on gender and age quota attributes.

During the data collection phase, surveyors established questionnaire sites at health service centers or relevant stations within the sampled communities, issuing paper or electronic recruitment notices and verifying respondents’ identities. Surveyors ensured that respondents met the inclusion criteria and did not fall under the exclusion criteria of the study. In instances where face-to-face surveys were impeded by COVID-19 restrictions, electronic questionnaires were distributed via instant messaging tools such as WeChat, with online video investigations conducted through Tencent Meeting, WeChat video, and other means. Each survey session lasted approximately 30–40 min, with the data collection phase spanning about 2 months.

**Figure 1:**
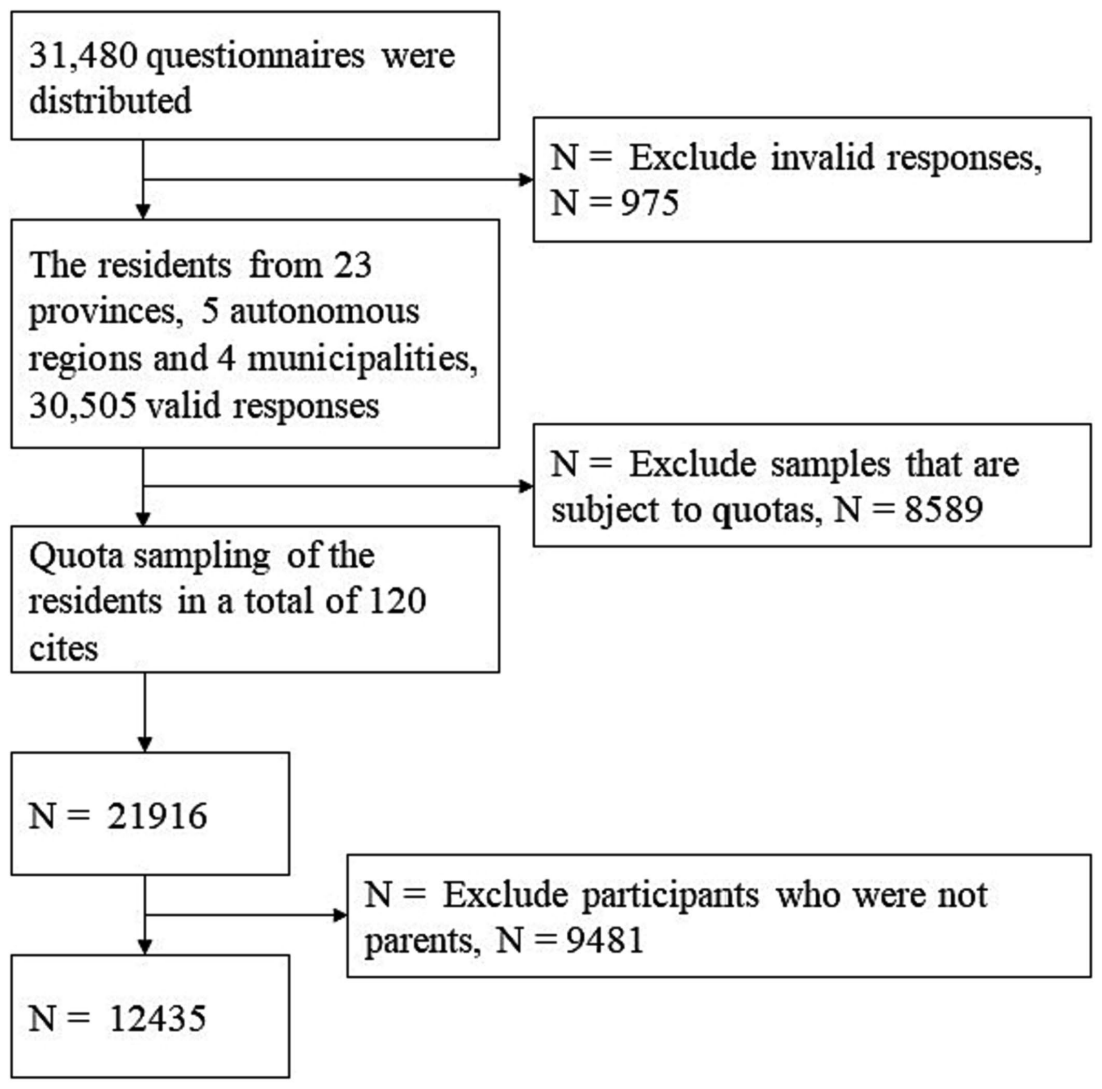
Flowchart of participant enrollment.

In total, 31,480 questionnaires were distributed, yielding 30,505 valid responses. After quota adjustments, the sample size amounted to 21,916 individuals. Following the exclusion of non-parent samples, 12,435 participants were included in the analysis of this study (Figure 1).

### 3.3 Instruments

The study questionnaire consisted of two parts. The first part was designed to capture the socio-demographic characteristics of the participants, including their parents, age, ethnicity, religious beliefs, place of residence, education level, marital status, average monthly household income, social network size, and neighborhood relations. The second part included three standardized scales: the Family Communication Scale (FCS), the short-form of the Family Health Scale (FHS-SF), and the Perceived Social Support Scale (PSSS).

#### 3.3.1 Demographic variables

The study examined the following demographic variables among the participants: parent (father and mother), age (18–59 years old or 60 years old and above), ethnicity (Han or ethnic minority), religion (religious beliefs or no religious beliefs), place of residence (urban and rural areas), education level (junior school or below, senior school or middle special school, junior college, and bachelor’s degree or above), average monthly household income (≤3,000 yuan, 3,001–6,000 yuan, 6,001–12,000 yuan, and ≥12,000 yuan), and marital status (unpartnered or partnered). To assess neighborhood relations, the study participants were asked to rate the quality of their relationships with their neighbors using a seven-point Likert scale ranging from 1 (very poor) to 7 (very good). To measure family social status, the participants were asked to rate their family’s social status on a seven-point scale from 1 (lowest level) to 7 (highest level). For social network size, the participants were asked to report the number of non-relatives they contacted on a given day in the past month through any means (i.e., telephone, email, and internet). Informal contact in this context referred to one-on-one contact with someone, whether known or unknown, and the response options included 0, 1–4, 5–9, 10–19, 20–49, 50–99, and ≥ 100, which were assigned values of 0, 1, 2, 3, 4, 5, 6, and 7, respectively.

#### 3.3.2 Family communication scale

Family communication is the third and facilitating dimension of the Circumplex Model of Marital and Family Systems Theory and focuses on the exchange of information, ideas, thoughts, and feelings among family members, with the goal of enhancing the cohesiveness of family members and addressing their developmental and situational needs. Olson’s Family Communication Scale (FCS) was employed to assess family communication (51). The scale consists of 10 items, rated on a five-point Likert scale, ranging from “strongly disagree” to “strongly agree,” with higher total scores indicating stronger family communication skills. In the present study, the FSC’s Cronbach’s alpha coefficient was 0.970, indicating a high level of internal consistency. Additionally, to validate the scale in the Chinese context, a Confirmatory Factor Analysis (CFA) was conducted. The CFA results indicated all items exhibited factor loadings above 0.8, demonstrating strong factor correlations. These results support the scale’s reliability and confirm its appropriateness for use within this specific population.

#### 3.3.3 Short-form of the family health scale

Researchers have widely used the Short Form of the Family Health Scale (FHS-SF) when evaluating family health; accordingly, the FHS-SF was also used to assess family health in this study. The scale was originally developed by Crandall and Weiss-Laxer et al. and later adapted into Chinese by Wang et al. (52) and Fei et al. (20). The FHS-SF is comprised of 10 items, which together measure four family health-related dimensions: Family/social/emotional health processes, Family healthy lifestyle, Family health resources, and Family external social supports. To rate their responses, participants used a five-point Likert scale ranging from “strongly disagree” to “strongly agree.” The Cronbach’s α coefficient for the FHS-SF in the present study was 0.822. Encouraging this result is comparable to that recorded in the original study conducted by Crandall et al. and indicates that the scale has good internal consistency. Overall, the FHS-SF has proven to be a reliable and valid tool for assessing family health that can be deployed in various cultures and settings.

#### 3.3.4 Perceived social support scale

The Perceived Social Support Scale (PSSS) is a reliable and valid tool that has been widely used to assess the perceived level of social support individuals receive from their social networks (53). To be precise, the PSSS focuses on the individual’s subjective perception of the support they receive from their family, friends, and others. The scale used in the study consists of three items that are rated on a seven-point Likert scale, ranging from “extremely disagree” to “extremely agree.” The PSSS has been validated in a number of studies and found to have consistency and accuracy when measuring perceived social support. A high PSSS score indicates a strong perception of social support; contrastingly, a low score suggests a lack of perceived support. In this study, the Cronbach’s α of PSSS was 0.885, indicating high internal consistency among the scale items. A Confirmatory Factor Analysis (CFA) was also performed to further validate the scale’s applicability in the Chinese context. The CFA results demonstrated a good fit, with a Comparative Fit Index (CFI) of 0.98, Root Mean Square Error of Approximation (RMSEA) of 0.07, and Standardized Root Mean Square Residual (SRMR) of 0.02. Additionally, all items exhibited factor loadings above 0.6, indicating strong factor correlations. These findings affirm the scale’s reliability for measuring social support in this study.

### 3.4 Statistical methods

The data were processed and analyzed using SPSS. Descriptive statistics were used to determine the number and proportion of categorical variables, along with the mean and standard deviation of continuous variables. For continuous variables, normality was assessed using skewness and kurtosis due to the large sample size, as Shapiro–Wilk and Kolmogorov–Smirnov tests are sensitive to sample size. In this study, the threshold for skewness and kurtosis was set at an absolute value of 1 and 7, respectively, to determine normality. The continuous variables showed the following skewness and kurtosis values: Family Communication (Skewness = −0.554, Kurtosis = 0.865), Family Health (Skewness = −0.682, Kurtosis = 2.36), Social Support (Skewness = −0.648, Kurtosis = 0.561), Social Network Size (Skewness = 0.285, Kurtosis = −1.43), and Neighborhood Relationships (Skewness = −0.884, Kurtosis = 0.839). These values suggest that the continuous variables were normally distributed, justifying the use of parametric tests in our regression analysis. In the present study, the demographic variables were treated as categorical variables and any differences in support for death education across the factors studied were compared using one-way ANOVA tests. Subsequently, a multiple linear stepwise regression model was employed to analyze the factors associated with predicting Chinese parents’ support for death education. This iterative process entailed constructing a regression model before removing potential explanatory variables in succession and testing for statistical significance after each iteration. In the regression analysis, the dependent variable was the support for death education, while the independent variables were as follows: age, parent, highest education, marital status, and religious belief at the individual level; family health, family communication, and average monthly household income at the family level; social support, neighborhood, and social network size at the societal level. Before proceeding with the regression model, the categorical variables were treated as dummy variables. Next, multicollinearity diagnostics were carried out using the variance inflation factor (VIF) and Tolerance. A VIF of less than 10 and a Tolerance of not less than 0.1 indicated that the independent variables in the model were not subject to severe multicollinearity. The associated test results are presented in Table 1. The R-squared represents the extent to which the dependent variable is explained by the independent variable and a *p* value of 0.05 or less was considered to be significant.

**Table 1:** The variance inflation factor of all variables in this study.

Categorical variables		Tolerance	VIF
Sex (ref: Male)			
	Female	0.989	1.011
Age		0.798	1.253
Religious beliefs (ref: No)			
	Yes	0.978	1.022
Highest educational (ref: Junior school or below)			
	Senior school or middle special school	0.795	1.257
	Junior college	0.758	1.318
	Bachelor’s degree or above	0.619	1.616
Average monthly household income		0.828	1.207
Marital status (ref: unpartnered)			
	Partnered	0.967	1.034
Family communication		0.54	1.852
Family health		0.623	1.606
Social support		0.69	1.45
Social network size		0.98	1.02
Neighborhood relationships		0.932	1.073

### 3.5 Ethics statement

This research project has been approved by the Shaanxi Institute of International Trade and Commerce, China (JKWH-2022-02). All the participants fully understood the study and voluntarily provided their consent (as documented on the informed consent forms).

## 4 Results

### 4.1 The statistical description of the SDE scores and influencing factors

Descriptive statistics for demographic variables and core variables are presented in Tables 2, 3. 12,435 subjects were included in this study, of whom 6,422 (51.6%) were mothers and 6,422 (51.6%) were fathers; 8,565 (68.9%) were aged 18–59 years and 3,870 (31.1%) were aged 60 years and over. There were 11,402 Han Chinese subjects (91.7%) and 1,033 ethnic minority subjects (8.3%); 653 religious subjects (5.3%) and 11,782 non-religious subjects (74.7%); 6,117 urban subjects (49.2%) and 6,318 rural subjects (6,318%). The number of subjects with secondary school education or below was 5,247, accounting for 42.2%, while the number of subjects with high school or secondary school education was 2,538, accounting for 20.4%; the number of subjects with college education was 1,771, accounting for 14.2%; the number of subjects with bachelor’s degree or above was 2,795, accounting for 22.5%; the number of subjects with average monthly household income below RMB 3,000; the number of subjects with average monthly household income below RMB 3,000 was 4,513, accounting for 36.3%; the number of subjects with average monthly household income between RMB 3,001 and 6,000 was 5,146, accounting for 41.4%; the number of subjects with average monthly household income between RMB6,001 and 12,000 was 2,050, accounting for 16.5%; the number of subjects with *per capita* monthly household income over RMB 12,000 was 726, accounting for 5.8%; the number of unpartnered subjects was 1,046 (8.4%); and the number of partnered subjects was 11,389 (91.6%).

**Table 2:** The statistical description of categorical variables and the scores of SDE.

Categorical variables		*N* (%)	The scores of SDE		
			M ± SD	t/F	*p* value
Total		12,435 (100)	65.51 ± 27.26	**-**	**-**
Sex					
	Father	6,013 (48.4)	64.35 ± 27.41	21.21	<0.001
	Mother	6,422 (51.6)	66.60 ± 27.08		
Age group					
	18–59 years old	8,565 (68.9)	65.98 ± 27.83	7.94	0.005
	60 years old and above	3,870 (31.1)	64.49 ± 25.92		
Minority group					
	Han nationality	11,402 (91.7)	65.53 ± 27.24	0.07	0.798
	Ethnic minorities	1,033 (8.3)	65.31 ± 27.48		
Religious beliefs					
	No	11,782 (94.7)	65.73 ± 27.23	14.09	<0.001
	Yes	653 (5.3)	61.62 ± 27.53		
Place of residence					
	Urban	6,117 (49.2)	67.28 ± 27.22	54	<0.001
	Rural	6,318 (50.8)	63.69 ± 27.18		
Highest educational					
	Junior school or below	5,247 (42.2)	63.26 ± 26.62	49.46	<0.001
	Senior school or middle special school	2,538 (20.4)	64.29 ± 27.21		
	Junior college	1,771 (14.2)	65.42 ± 28.44		
	Bachelor’s degree or above	2,795 (22.5)	70.81 ± 27.02		
Average monthly household income					
	≤3,000	4,513 (36.3)	62.21 ± 27.64	48.75	<0.001
	3,001–6,000	5,146 (41.4)	66.08 ± 26.83		
	6,001–12,000	2,050 (16.5)	69.06 ± 26.45		
	>12,000	726 (5.8)	72.04 ± 27.55		
Partner status					
	Unpartnered	1,046 (8.4)	60.76 ± 28.80	5.94	0.015
	Partnered	11,389 (91.6)	65.69 ± 27.13		

SDE, Support for death education.

**Table 3:** The statistical description of metric variables.

Metric variables	M ± SD
Family communication	38.08 ± 7.51
Family health	35.91 ± 6.53
Social support	15.03 ± 3.68
Social network size	2.90 ± 2.22
Neighborhood relationships	5.85 ± 1.47

The participants who were mothers (66.60 ± 27.08), aged 18–59 years old (65.98 ± 27.83), had no religious beliefs (65.73 ± 27.23), lived in urban areas (67.28 ± 27.22), had a higher level of education, had a higher average monthly household income and had a history of marriage, had a higher level of support for death education.

### 4.2 The factors relevant to the support for death education

The factors relevant to Chinese parents’ support for death education are detailed in Table 4. The stepwise regression analysis resulted in an *R*^2^ value of 0.254, with an adjusted *R*^2^ of 0.08, indicating the proportion of variance in the dependent variable that is predictable from the independent variables. The VIF values for the variables in this stepwise regression can be found in Table 5, which demonstrates that the VIF values for all variables are less than 10, indicating that the model does not suffer from any serious issues of multicollinearity. With regard to individual-level factors in the social ecological theory model, the results indicate that Chinese parents’ support for death education was positively predicted by parent (*β* = 0.04) and highest education (*β* = 0.07), but religious belief (*β* = −0.02) had a negative impact. As for family-level factors, average monthly household income (*β* = 0.07), family communication (*β* = 0.02) and family health (*β* = 0.03) were significant positive predictors of Chinese parents’ support for death education. At the social level, the results show that social support (*β* = 0.15), neighborhood relationship (*β* = 0.11), and social network size (*β* = 0.05) were all significant positive predictors of Chinese parents’ support for death education.

**Table 4:** The stepwise regression analysis of factors associated with the support for death education.

Variables	*β*	SE	*t*	*p*
Sex (Ref: Father)				
Mother	0.04	0.47	5.08	<0.001
Highest education	0.07	0.61	7.94	<0.001
Religious beliefs				
Yes	−0.02	1.06	−2.49	0.027
Average monthly household income	0.07	0.29	8	<0.001
Family health	0.03	0.04	2.46	0.015
Family communication	0.02	0.04	2.21	0.041
Social support	0.15	0.08	14.03	<0.001
Neighborhood relationship	0.11	0.17	11.78	<0.001
Social network size	0.05	0.11	5.96	<0.001

**Table 5:** The VIF values for the variables in this stepwise regression.

Categorical variables		Tolerance	VIF
Sex (ref: Father)			
	Mother	0.988	1.013
Age group (ref: 15–59 years old)			
	60 years old and above	0.799	1.252
Minority group (ref: Han nationality)			
	Ethnic minorities	0.915	1.093
Religious beliefs (ref: No)			
	Yes	0.905	1.105
Place of residence (ref: Urban)			
	Rural	0.776	1.289
Highest educational level (ref: Junior school or below)			
	Senior school or middle special school	0.758	1.319
	Junior college	0.709	1.409
	Bachelor’s degree or above	0.578	1.731
*Per capita* monthly household income, yuan (ref: ≤3,000)			
	3,001–6,000	0.715	1.398
	6,001–12,000	0.713	1.402
	>12,000	0.815	1.226
Marital status (ref: Unmarried)			
	Married	0.167	5.970
	Divorced	0.333	3.007
	widowed	0.235	4.255
Family communication		0.538	1.860
Family health		0.621	1.611
Family social status		0.755	1.325
Social support		0.691	1.448
Social network size		0.979	1.021
Neighborhood relationships		0.737	1.356

## 5 Discussion

This study drew on society ecosystems theory to analyze the extent to which the micro-individual, mezzo-family, and macro-society levels influence Chinese parents’ support for death education. Based on the results, the following factors were found to influence parental support for death education.

At the micro-individual level, parent (H2), education level (H3), and religious belief (H5) positively influenced Chinese parents’ support for death education. It was found that the more educated a Chinese parent is, the more they are likely to support the provision of death education. Notably, support for death education was higher among highly educated, religious women.

At the mezzo-family level, average monthly household income (H6), family health (H7), and family communication (H8) were positively correlated with Chinese parents’ support for death education. The higher the family’s average monthly household income, the healthier it is, and the better it is at communication, the more supportive the parents in the family are of death education.

At the macro-society level, social support (H9), neighborhood relationships (H10), and social network scale (H11) were positively correlated with Chinese parents’ support for death education. The stronger the social support, the more harmonious the neighborhood relationship, and the larger the social network scale, the more supportive the parents will be of death education. Of the 11 factors assessed in this study, the influences of social support and neighborhood relationships on support were the most significant.

### 5.1 The micro-individual level

At the micro-individual level, age, parent, highest education, and religious belief were the leading factors influencing Chinese parents’ support for death education.

#### 5.1.1 Parent

The study found that parent has an important impact on Chinese parents’ support for death education, which is consistent with existing research findings. Specifically, one study showed that the demand for death education is higher among female students than their male peers (29), while another observed that female adolescents are more likely to include death education in the curriculum than males (54). These studies suggest that parents’ differences play an important role in the extent to which an individual supports death education. Men and women differ in their social roles, values, and emotional expression, which can lead to different responses to death-related issues, such as their attitudes, behaviors, and understanding. Therefore, among Chinese parents, mothers are more likely to support death education.

#### 5.1.2 Highest education

This study also found that Chinese parents’ support for death education varies significantly depending on their education level. Again, this conclusion is consistent with existing research. A study of Spanish parents by Molina-Ortiz et al. concluded that educational background was positively correlated with attitudes toward hospice care and death education (55). Elsewhere, in their study of British parents by Sallnow et al. (56), educational background was shown to be positively correlated with parents’ willingness to talk about death with their children. The present study’s results indicate that better-educated Chinese parents (i.e., those who have completed higher levels of education) are more supportive of death education than those with a junior high school education or below. This may be because highly educated parents have more diverse knowledge, are more open to thinking and accepting views on death education in different cultures and values, and are more willing to expose themselves and their children to relevant information.

#### 5.1.3 Religious belief

In the present study, religious belief impacted Chinese parents’ support for death education, in that parents with religious beliefs were less likely to support the provision of death education. Encouragingly, this result is consistent with the existing research. When the content of death education conflicts with the parent’s strong religious beliefs, they may reject death education (57, 58). One possible reason for this observation is that religious beliefs often offer explanations for the unknown, including death. For example, in those religions that offer a specific view of the afterlife, this can provide a sense of security about death. Therefore, religious parents may be less supportive of death education for their children because they fear that it takes away the security that comes with religion. It is worth noting that in a few cases, some people with more strongly religious beliefs may be more supportive of death education, especially if it broadly conforms to their beliefs and values (37).

### 5.2 The mezzo-family level

At the mezzo family level, average monthly household income, family health status, and family communication are positively correlated with Chinese parents’ support for death education.

#### 5.2.1 Average monthly household income

This study found that the average monthly household income was positively correlated with parents’ support for death education. Again, this finding is consistent with the existing research. Household income is impacted by a range of factors, including but not limited to the parents’ educational background, social status, and values. Studies have shown that parents with different household incomes exhibit different attitudes and levels of support for their children’s education (59, 60). These differences may have implications for how parents choose to raise their children and approach the topic of death with them. Therefore, parents with a higher average monthly household income tend to be more willing to provide their children with psychological and behavioral support pertaining to death topics, which is reflective of a higher degree of support for death education.

#### 5.2.2 Family health status

The results of this study showed that the healthier the family, the more supportive the parents are of death education, which aligns with the existing research results. In other studies, improving family health has been shown to enhance parents’ understanding and acceptance of death, thereby allowing them to recognize and attach more importance to death education (61). Since the family environment is the environment in which individuals first face death, communication, and support among family members play a very important role in dealing with the emotions and perceptions of death (61, 62). In healthier families, parents who communicate with their children, offering respect and encouragement, are more willing to help their children improve their understanding of death through death education.

#### 5.2.3 Family communication

In the present study, positive family communication was found to increase parental support for death education, which is consistent with existing research. For example, in a sample of Portuguese adolescents, Abreu et al. observed an association between positive family communication and higher death awareness and lower death anxiety (63). This may be because effective family communication reduces the stigma and fear associated with death, while also promoting family members’ understanding of death and coping skills. When family communication is open and supportive, parents are more likely to discuss death and support death education; by way of contrast, in those families that fail to communicate effectively, avoid sensitive topics, or experience a lack of trust, parents may be less supportive of death education.

### 5.3 The macro-social level

#### 5.3.1 Social support

This study showed that in China, the stronger the perceived degree of social support an individual enjoys, the more supportive parents were of death education. This conclusion is consistent with existing research: Otsuka et al. (64) identified an association between instrumental social support and emotional social support with a reduced risk of suicide. Jiang’s study showed that psychological support had a prominent positive predictive effect on death education demand and neutral death attitude (65). On this basis, it is suggested that improving social support can facilitate Chinese parents forming a positive and healthy attitude toward death and increase their demand and support for death education (note that demand in this context can also be seen as this support).

#### 5.3.2 Neighborhood relationships

Positive neighborhood relationships were shown to promote support for death education. This aligns with the findings of the existing literature, where a number of studies have found that community or neighborhood relationships can affect mortality (66–68). This may be because a discordant neighborhood leads to less communication about death, fewer effective interventions, and limited consensus. Harmonious neighborhood relationships can expose children and parents to death, in addition to providing parents with emotional comfort, advice, and practical help, all of which are conducive to promoting a positive attitude toward death education.

#### 5.3.3 Social network scale

The larger the social network, the more supportive parents were found to be of death education. This conclusion is consistent with existing research. For example, Yoo-Jeong et al. (69) determined that the social network scale was related to the quality of life, which in turn affects attitudes toward death. Doka et al. (70) concluded that participation in social networks allows people to be more open and talk more freely about death. This may be because their social network scale affects their chances of gaining perspective on death and learning from others. Accordingly, a larger social network might provide Chinese parents with a broader perspective on death education and increase their support for its provision.

### 5.4 Research value and limitations

This study is the first of its kind to explore the influencing factors of Chinese parents’ support for death education based on a nationwide sample of Chinese parents. Notably, this study offers research value in theory and practice. First, this study attends to the gap in examining the factors influencing the support for death education from the perspective of parents. Secondly, it expands the society ecosystems theory from the perspective of death education. Finally, this study offers references and feasible suggestions that the Chinese local and national governments can use to formulate public policies to draw the public’s attention to death education. Additionally, it also promotes the popularization of death education courses in primary and secondary schools and universities and reduces the suicide rate of adolescents.

However, there are some limitations to this study: as the nationwide sample is cross-sectional data, it is not possible to study relevant changes over time. Additionally, the regression model’s explanatory power is somewhat limited, as indicated by an *R*^2^ value of 0.254. This suggests that a considerable portion of the variance in the support for death education remains unexplained, possibly due to unaccounted factors or the complex nature of the subject matter. Since self-reported information and self-assessment scales are used in the study, this may lead to the introduction of reporting bias. In addition, it cannot be determined how many participants viewed the questionnaire but did not complete it; as such, it is not possible to assess non-response bias. Similarly, it was not possible to exclude those parents who are learning about death education. Given these considerations, it is recommended that future research should account for the possibility of measurement bias and the limited explanatory power of statistical models. Longitudinal studies could be particularly valuable in understanding the evolving dynamics and contributing factors to Chinese parents’ support for death education, providing a more comprehensive understanding of this complex issue.

Future studies could build on the current research to explore a broader range of factors influencing Chinese parents’ support for death education, such as personality characteristics and social cultures. Meanwhile, longitudinal studies could be carried out to assess how and to what extent various factors influence Chinese parents’ attitudes to death education. At the same time, the Chinese government should strengthen its publicity and education efforts to raise public awareness of and support for family health and family communication, specifically targeting men, young people, people with low educational attainment, single people, and those without religious beliefs. In addition, active steps should be taken to increase residents’ income, carry forward the good-neighborly approach to living, and promote friendly traditional culture. It is necessary to advance the cause of death education in schools, improve various social support measures for death education, and help residents form a correct view of death.

## 6 Conclusion

In the present study, Chinese parents’ support for death education was influenced by multiple factors at individual, family, and social levels. Specifically, social support and neighborhood relationships had the most significant influence on support. Based on these findings, it is suggested that development planning, publicity, and other relevant government departments, as well as public welfare groups, strengthen the promotion of death education, provide more appropriate social support and promote neighborhood harmony. In addition, parents’ education level and average monthly household income also exerted a notable impact on support. The government should further increase access to higher education and actively promote the growth of residents’ income as part of its efforts to develop death education.

In addition, the study found that social network scale, family health, family communication, and age were all positively correlated with Chinese parents’ support for death education. At the same time, family education and death-related experiences were also shown to influence death education. Parent and religious belief impacted support in highly different ways. Therefore, there is a need to increase access to death education for women, while simultaneously avoiding the influence of religion on parents.

## Data availability statement

The original contributions presented in the study are included in the article/supplementary material, further inquiries can be directed to the corresponding authors.

## Ethics statement

The study approval was obtained from the Clinical Research Ethics Committee of the Second Xiangya Hospital of Central South University. The ethics number is: No.2022-K050. The studies were conducted in accordance with the local legislation and institutional requirements. The participants provided their written informed consent to participate in this study.

## Author contributions

HC: Conceptualization, Methodology, Writing – original draft, Writing – review & editing. YX: Conceptualization, Funding acquisition, Writing – original draft, Writing – review & editing. XH: Formal analysis, Methodology, Software, Writing – original draft, Writing – review & editing. SF: Investigation, Writing – original draft, Writing – review & editing. HW: Investigation, Validation, Writing – original draft, Writing – review & editing. LL: Formal analysis, Visualization, Writing – original draft, Writing – review & editing. YW: Resources, Writing – original draft, Writing – review & editing.
